# Supplementary material for: The first ice-free day in the Arctic Ocean could occur before 2030
Source: Nat Commun. 2024 Dec 3;15:10101. doi: 10.1038/s41467-024-54508-3 (PMC11615213; doi:10.1038/s41467-024-54508-3)
Supplement: Supplementary file 1 — Supplementary Information [file 41467_2024_54508_MOESM1_ESM.pdf]

Supplementary Information for "The first ice-free day in the Arctic Ocean could occur before 2030"

Céline Heuzé[1*†] and Alexandra Jahn[2*†]

[1*]Department of Earth Sciences, University of Gothenburg, Box 460, 405 30, Göteborg, Sweden.
[2*]Department of Atmospheric and Oceanic Sciences and Institute of Arctic and Alpine Research, University of Colorado at Boulder, Boulder, CO, USA.

*Corresponding author(s). E-mail(s): celine.heuze@gu.se; alexandra.jahn@colorado.edu;
†These authors contributed equally to this work.

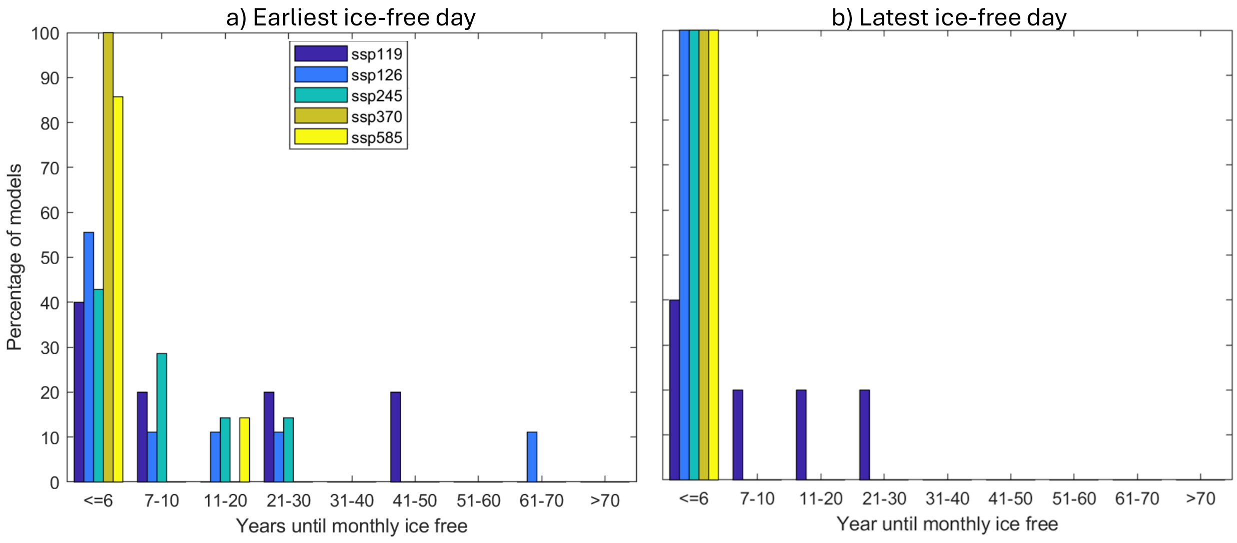

Fig. S1 Time from daily to monthly ice-free conditions: For the earliest (a) and latest (b) simulations shown on Fig. 1a and b, respectively, histograms of the time from the first ice-free day to the first ice-free month in the same ensemble member. For models that only had one ensemble member available, the same ensemble member is shown in the earliest and latest histograms. This figure shows that there is some scenario dependence for the earliest ensemble members, where the higher emission scenario leads to short times between the first ice-free day and first ice-free month, but none for the latest ensemble member, aside from the lowest emission scenario where there is a longer delay for some models while all other scenarios show the first ice-free month within less than 6 years of the first ice-free day. SSP stands for Shared Socioeconomic Pathway.

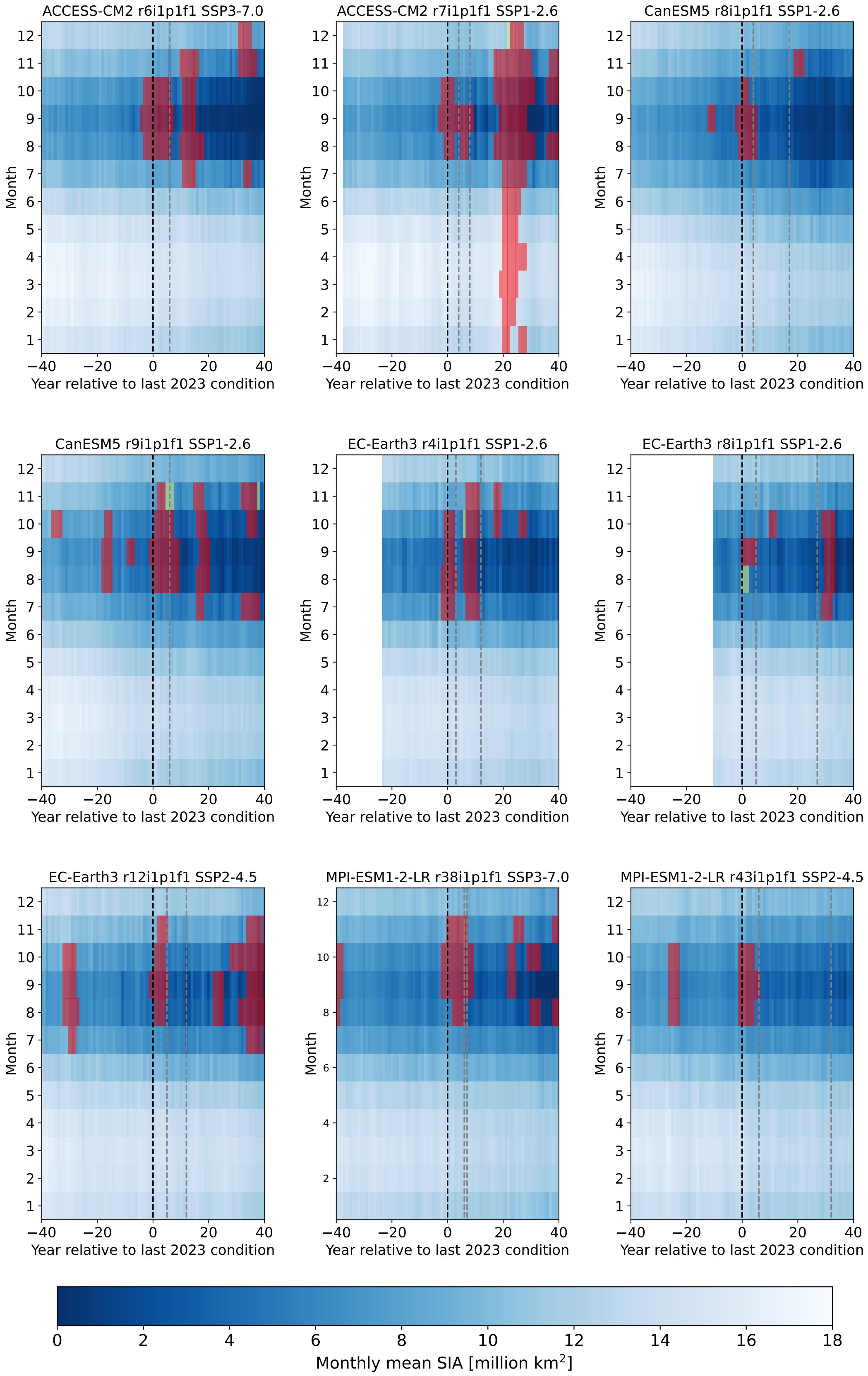

Fig. S2 Rapid Ice Loss Events (RILEs) on the way to the first ice-free day: The monthly mean sea ice area (SIA) from each quick transition simulation for all months of the year is shown in blue shading [in million km^2], with RILEs in a given month overlaid in red. RILE events in each month of the year are defined based on monthly mean sea ice extent [1], as described in the Methods section 4. When slightly relaxing the RILE criteria from a trend of -0.3 million km^2 per year to -0.299 million km^2 per year, additional RILEs show up for some simulations (shown in yellow). Vertical dashed grey lines indicate the year of the first ice-free day and first ice-free month. When only one grey line is shown then the first ice-free day and month occur in the same year. The vertical dashed black line shows the 2023 equivalent year. This figure shows that all quick transition members have a RILE or near RILE in August and September during the transition from the 2023 equivalent year to the first ice-free day, with some simulations also showing RILES in additional months.

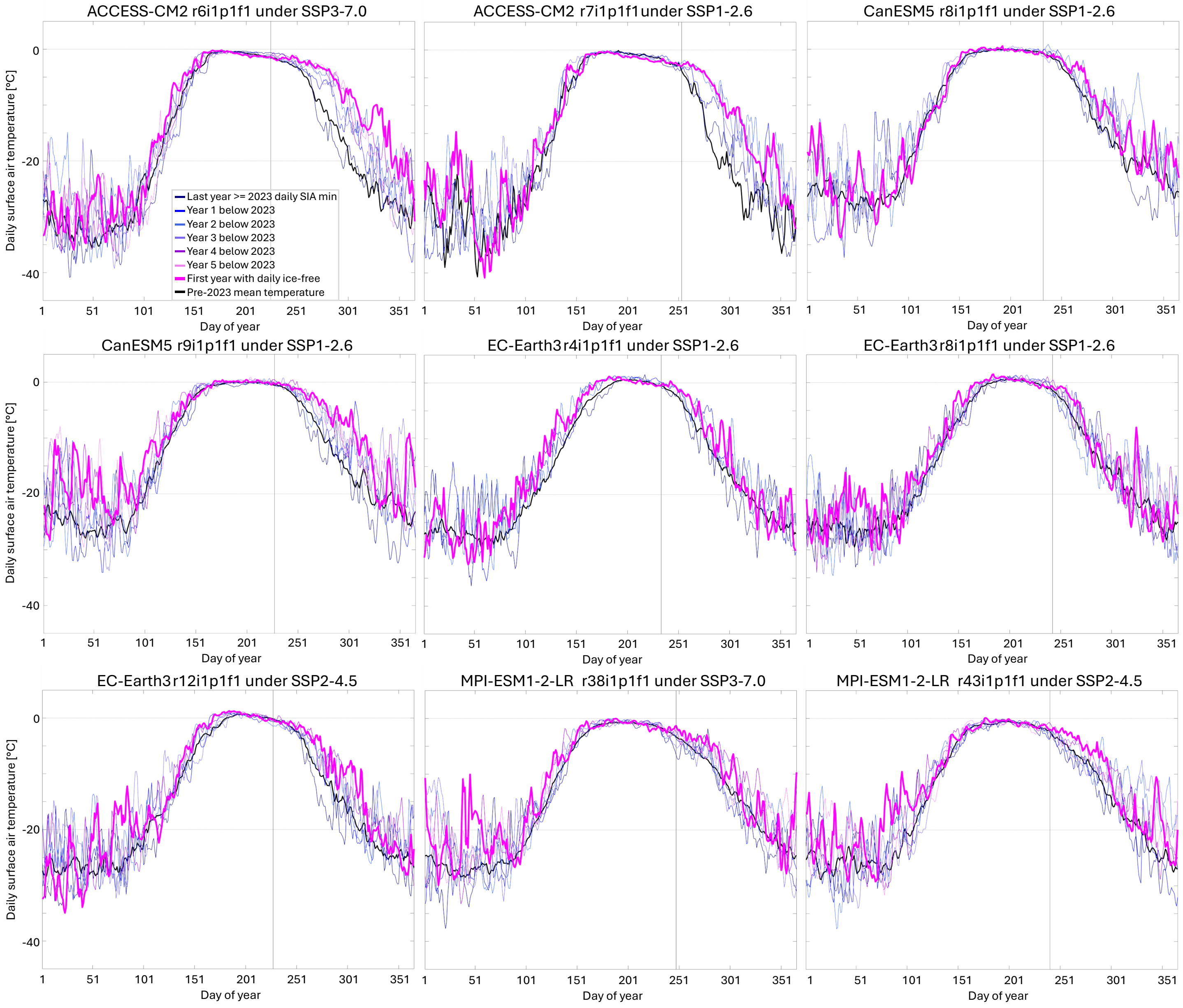

Fig. S3 Surface air temperature on the way to first daily ice-free conditions: As in Figure 3, but for the daily average surface air temperature north of 80°N, from the last year the daily sea ice area (SIA) minimum was above to or equal to the 2023 daily SIA minimum (in dark blue) to the first year where the daily SIA goes ice-free (bold line in pink, see legend, same color coding as in Figure 3 for the years that are shown in both figures). Black line is the pre-2023 average. This shows that the last year was unusually warm, for all cases.

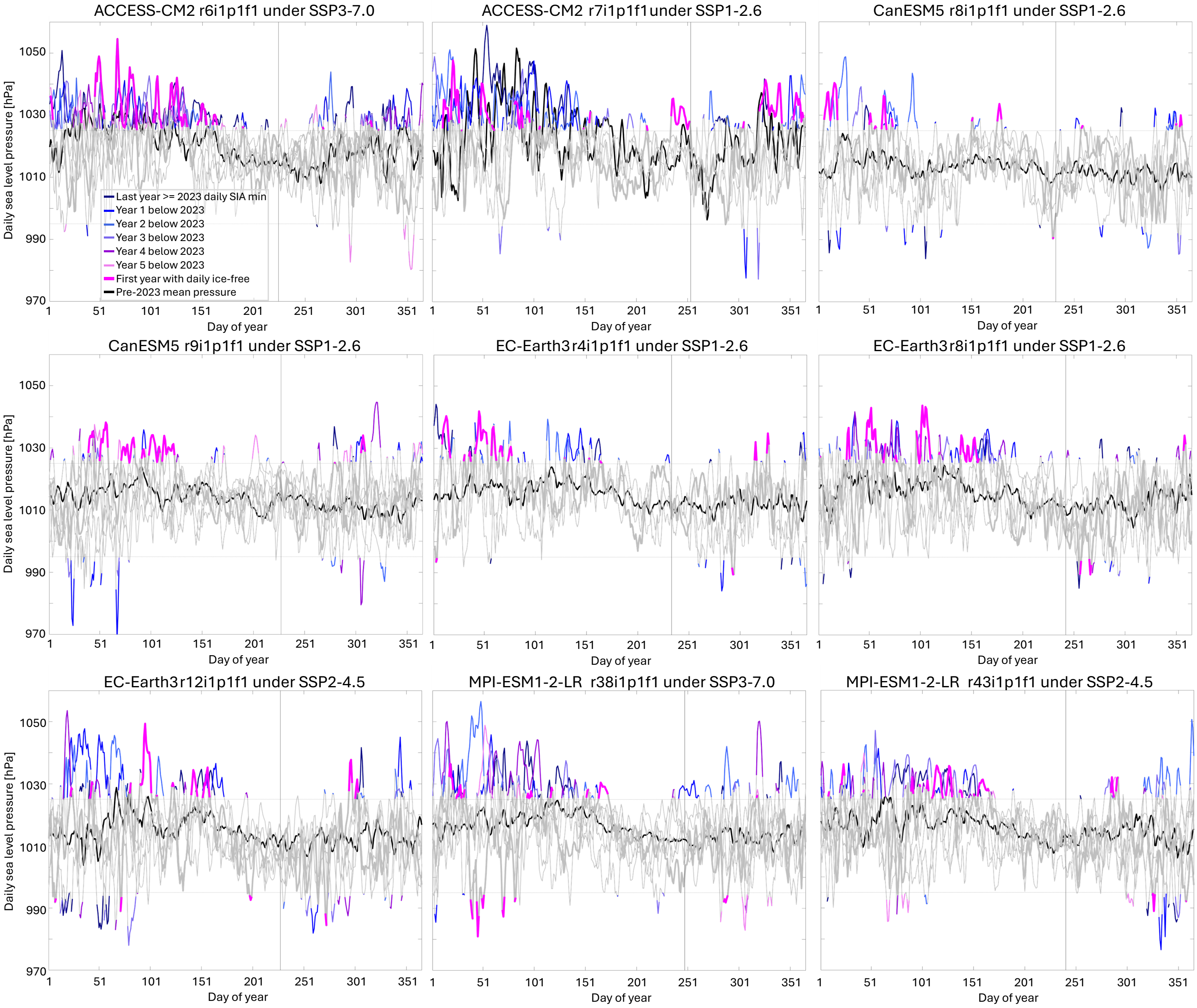

Fig. S4 Sea level pressure on the way to first daily ice-free conditions: As in Figure 3, but for the daily average sea level pressure north of 80°N, from the last year the daily SIA minimum was above to or equal to the 2023 daily sea ice area (SIA) minimum (in dark blue) to the first year where the daily SIA goes ice-free (bold line in pink, see legend, same color coding as in Figure 3 for the years that are shown in both figures). Black line is the pre-2023 average. Only extreme events are coloured; the vertical thick grey line indicates the first ice-free day. This shows that the last year was mostly high-pressure dominated in winter and spring (blocking events), for all cases, but also had strong storms, leading to thermo- and dynamic stresses to the sea ice.

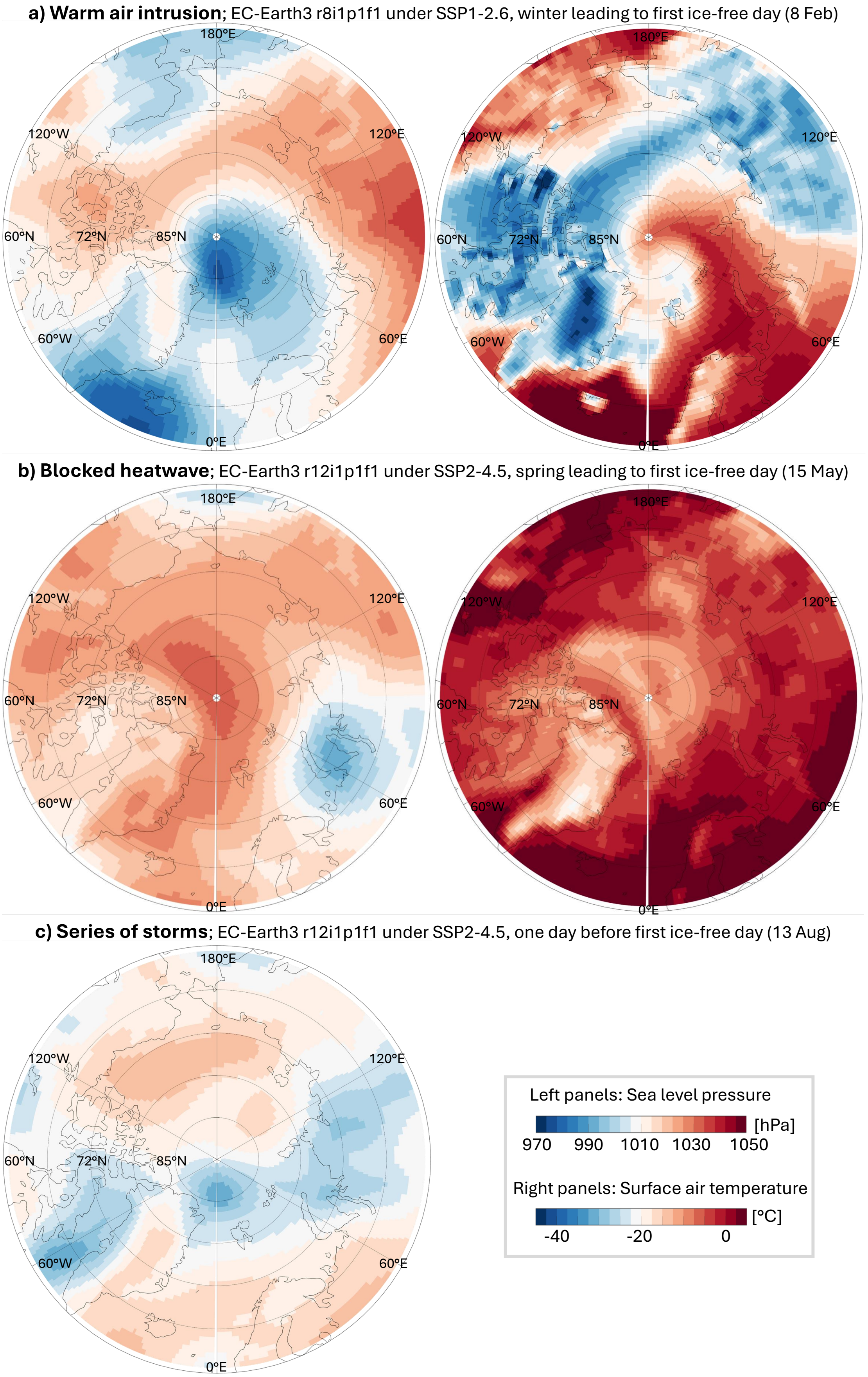

Fig. S5 Atmospheric events leading to an ice-free day: Sea level pressure (left) and when relevant, surface air temperature (right) on exemplary days of the last year before the model had its first ice-free day illustrating a) a warm air intrusion; b) a blocking pattern coinciding with a heatwave; and c) a series of a least four storms.

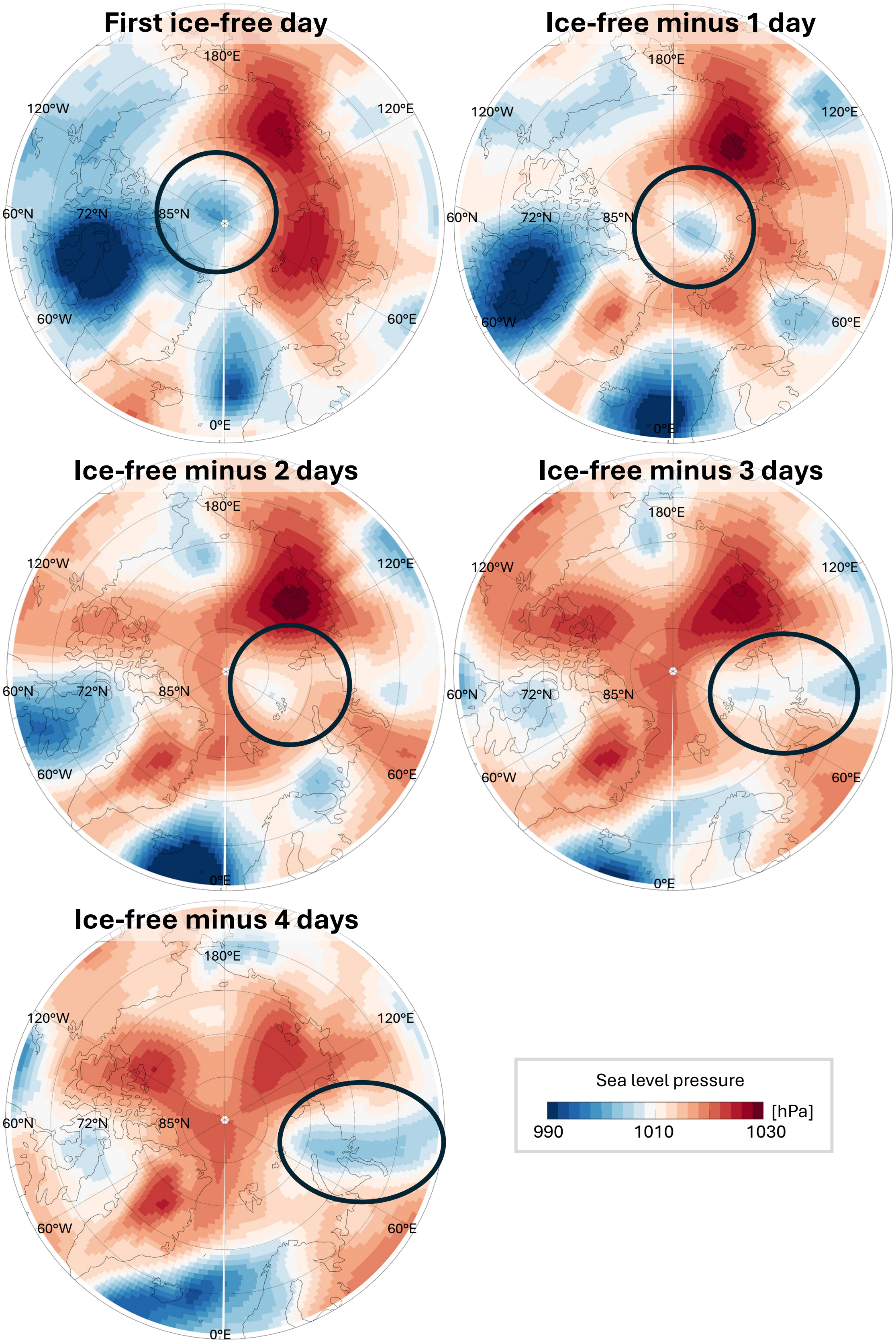

Fig. S6 The fastest ice-loss simulation and its last storm: Sea level pressure, backtracking from the first ice-free day a storm crossing the Arctic for our fastest case, EC-Earth3 r4i1p1f1 under SSP1-2.6.

Model	SSP1-1.9		SSP1-2.6		SSP2-4.5		SSP3-7.0		SSP5-8.5	
ACCESS-CM2	-		r7i1p1f1<br>r6i1p1f1<br>difference	4<br>23<br>19	r1i1p1f1<br>r4i1p1f1<br>difference	9<br>20<br>11	r6i1p1f1<br>r3i1p1f1<br>difference	6<br>21<br>15	r9i1p1f1<br>r4i1p1f1<br>difference	8<br>22<br>14
BCC-CSM2-MR	-		r1i1p1f1	54	r1i1p1f1	20	-		r1i1p1f1	28
CanESM5	r4i1p1f1<br>r5i1p1f1<br>difference	18<br>34<br>16	r8i1p1f1<br>r1i1p2f1<br>difference	4<br>28<br>24	r8i1p1f1<br>r5i1p2f1<br>difference	8<br>27<br>19	r7i1p1f1<br>r5i1p2f1<br>difference	9<br>22<br>13	r5i1p2f1<br>r10i1p2f1<br>difference	9<br>21<br>12
CNRM-CM6-1-HR	-		r1i1p1f2	>70	-		-		r1i1p1f2	10
EC-Earth3	r4i1p1f1	18	r4i1p1f1<br>r1i1p1f1<br>difference	3<br>7<br>4	r12i1p1f1<br>r16i1p1f1<br>difference	5<br>21<br>16	-		-	
IPSL-CM5A2-INCA	-		r1i1p1f1	>70	-		r1i1p1f1	18	-	
MIROC6	r1i1p1f1	52	r3i1p1f1<br>r1i1p1f1<br>difference	20<br>35<br>15	r1i1p1f1<br>r3i1p1f1<br>difference	22<br>39<br>17	r2i1p1f1<br>r3i1p1f1<br>difference	15<br>27<br>12	r3i1p1f1<br>r1i1p1f1<br>difference	23<br>27<br>4
MIROC-ES2L	r1i1p1f2<br>r10i1p1f2<br>difference	24<br>59<br>35	-		-		-		-	
MPI-ESM-1-2-HAM	-		-		-		r2i1p1f1<br>r1i1p1f1<br>difference	13<br>19<br>6	-	
MPI-ESM1-2-LR	r41i1p1f1<br>r50i1p1f1<br>difference	36<br>>70<br>34	r5i1p1f1<br>r48i1p1f1<br>difference	12<br>>70<br>58	r43i1p1f1<br>r18i1p1f1<br>difference	6<br>46<br>40	r38i1p1f1<br>r1i1p1f1<br>difference	6<br>48<br>42	r7i1p1f1<br>r44i1p1f1<br>difference	9<br>35<br>26
NorESM2-LM	-		r1i1p1f1	>70	r2i1p1f1<br>r3i1p1f1<br>difference	24<br>36<br>12	r1i1p1f1<br>r3i1p1f1<br>difference	38<br>>70<br>32	r1i1p1f1	23

Table S1 Time to the first ice-free day: For each selected CMIP6 model and for each scenario (Shared Socioeconomic Pathway SSP), the ensemble members with the earliest (top) and latest (bottom) ice-free day, years until that ice free day, and width of the distribution (difference between earliest and latest). Only one value is shown if the model had only one ensemble member available. The number of available ensemble members for each model is shown in Supp. Tab. S5. If the entry is >70 years, this means that the simulation did not reach ice-free conditions before the end of the 21st century simulations.

Model	SSP1-1.9		SSP1-2.6		SSP2-4.5		SSP3-7.0		SSP5-8.5	
ACCESS-CM2	-		r9i1p1f1<br>r10i1p1f1<br>difference	7<br>26<br>19	r2i1p1f1<br>r4i1p1f1<br>difference	10<br>20<br>10	r6i1p1f1<br>r4i1p1f1<br>difference	6<br>22<br>16	r9i1p1f1<br>r4i1p1f1<br>difference	8<br>22<br>14
BCC-CSM2-MR	-		r1i1p1f1	54	r1i1p1f1	21	-		r1i1p1f1	31
CanESM5	r4i1p1f1<br>r5i1p1f1<br>difference	18<br>34<br>16	r9i1p1f1<br>r1i1p2f1<br>difference	6<br>32<br>26	r8i1p1f1<br>r5i1p2f1<br>difference	8<br>28<br>20	r7i1p1f1<br>r10i1p1f1<br>difference	11<br>29<br>18	r6i1p1f1<br>r1i1p1f1<br>difference	11<br>22<br>11
CNRM-CM6-1-HR	-		r1i1p1f2	>70	-		-		r1i1p1f2	10
EC-Earth3	r4i1p1f1	27	r1i1p1f1<br>r8i1p1f1<br>difference	8<br>26<br>18	r10i1p1f1<br>r16i1p1f1<br>difference	7<br>22<br>15	-		-	
IPSL-CM5A2-INCA	-		r1i1p1f1	>70	-		r1i1p1f1	18	-	
MIROC6	r1i1p1f1	>70	r2i1p1f1<br>r3i1p1f1<br>difference	25<br>48<br>23	r1i1p1f1<br>r3i1p1f1<br>difference	23<br>42<br>19	r2i1p1f1<br>r3i1p1f1<br>difference	15<br>27<br>12	r3i1p1f1<br>r2i1p1f1<br>difference	24<br>29<br>5
MIROC-ES2L	r1i1p1f2<br>r3i1p1f2<br>difference	28<br>>70<br>>42	-		-		-		-	
MPI-ESM-1-2-HAM	-		-		-		r2i1p1f1<br>r1i1p1f1<br>difference	13<br>20<br>7	-	
MPI-ESM1-2-LR	r48i1p1f1<br>r50i1p1f1<br>difference	48<br>>70<br>>22	r34i1p1f1<br>r9i1p1f1<br>difference	21<br>>70<br>>49	r26i1p1f1<br>r18i1p1f1<br>difference	15<br>46<br>31	r38i1p1f1<br>r1i1p1f1<br>difference	7<br>48<br>41	r7i1p1f1<br>r42i1p1f1<br>difference	9<br>40<br>31
NorESM2-LM	-		r1i1p1f1	>70	r1i1p1f1<br>r2i1p1f1<br>difference	32<br>42<br>11	r1i1p1f1<br>r3i1p1f1<br>difference	41<br>>70<br>>29	r1i1p1f1	24

Table S2 Time the first ice-free month: Same as Supp. Tab. S1 but for the first ice-free month.

Simulation	Autumn (275 - 365)			Winter (1 - 100)			Spring (101 - 150)			Summer (151 - 225)		
	SLP 10%	90%	HDD	SLP 10%	90%	HDD	SLP 10%	90%	HDD	SLP 10%	90%	HDD
ACCESS-CM2 r6i1p1f1 SSP370	-12.3	-8.4	86	-2.4	5.9	47	5.3	4.8	152	0.2	1.7	2
ACCESS-CM2 r7i1p1f1 SSP126	-5.2	-9.8	165	-6.3	-3.7	82	4.3	-9.2	25	-5.0	-3.9	-24
CanESM5 r8i1p1f1 SSP126	-3.7	-8.9	165	-2.8	-4.9	33	3.6	-9.9	-50	-4.1	-5.2	13
CanESM5 r9i1p1f1 SSP126	-1.4	-5.9	261	-2.1	-3.2	289	5.7	-7.2	70	-4.6	-7.2	-1
EC-Earth3 r4i1p1f1 SSP126	-2.4	-3.0	138	5.8	4.5	-36	3.0	-7.7	72	-3.4	0.4	21
EC-Earth3 r8i1p1f1 SSP126	2.3	0.9	128	4.3	4.3	184	13.9	10.2	74	-4.7	-1.7	44
EC-Earth3 r12i1p1f1 SSP245	-7.8	-4.2	262	6.3	-3.1	21	-4.2	2.8	-3	-2.3	0.7	46
MPI-ESM1-2-LR r38i1p1f1 SSP370	-8.0	-3.8	118	7.4	-4.4	216	-4.3	1.7	81	-1.8	-0.9	-2
MPI-ESM1-2-LR r43i1p1f1 SSP245	-7.0	-3.0	64	8.2	-3.3	322	-3.8	2.4	57	-2.4	-1.1	6

Table S3 How unusual the year with the first ice-free day was: Difference between the year with the first ice-free day and the years prior since the 2023 equivalent year for each season (day of year indicated in brackets) in the 10th and 90th percentiles in sea level pressure (SLP, in hPa) and heating degree days (HDD) north of 80°N. The 10th and 90th SLP percentiles indicate extremely low and high pressures, respectively, while HDD is used here as a proxy for heat waves. Typical standard deviations across the models are 5 hPa and up to 3°C.

Simulation	Barents	Kara	Laptev	East Sib.	Chukchi
ACCESS-CM2 r6i1p1f1 SSP370	-32	65	18	14	20
ACCESS-CM2 r7i1p1f1 SSP126	71	-31	-50	11	-8
CanESM5 r8i1p1f1 SSP126	-40	-140	-105	-18	53
CanESM5 r9i1p1f1 SSP126	-35	-8	87	28	41
EC-Earth3 r4i1p1f1 SSP126	125	-19	-99	-80	-12
EC-Earth3 r8i1p1f1 SSP126	84	-45	-60	32	84
EC-Earth3 r12i1p1f1 SSP245	67	75	21	-30	-2
MPI-ESM1-2-LR r38i1p1f1 SSP370	6	80	-43	-12	10
MPI-ESM1-2-LR r43i1p1f1 SSP245	-20	49	107	-10	-14

Table S4 How unusual the summer with the first ice-free day was (continued): Difference between the year with the first ice-free day and the years prior since the 2023 equivalent year for summer only (day of year 151 to 225 as per Supp. Tab. S3) in heating degree days (HDD) for the shelf seas of the Arctic: Barents Sea, Kara Sea, Laptev Sea, East Siberian Sea (East Sib.), and Chukchi Sea.

Model name and experiment	data doi and citation
ACCESS-CM2 piControl (1)	10.22033/ESGF/CMIP6.4311 [2]
ACCESS-CM2 historical (10)	10.22033/ESGF/CMIP6.4271 [3]
ACCESS-CM2 SSP1-2.6 (10)	10.22033/ESGF/CMIP6.4319 [4]
ACCESS-CM2 SSP2-4.5 (10)	10.22033/ESGF/CMIP6.4322 [5]
ACCESS-CM2 SSP3-7.0 (9)	10.22033/ESGF/CMIP6.4323 [6]
ACCESS-CM2 SSP5-8.5 (10)	10.22033/ESGF/CMIP6.4332 [7]
BCC-CSM2-MR historical (1)	10.22033/ESGF/CMIP6.2948 [8]
BCC-CSM2-MR SSP1-2.6 (1)	10.22033/ESGF/CMIP6.3028 [9]
BCC-CSM2-MR SSP2-4.5 (1)	10.22033/ESGF/CMIP6.3030 [10]
BCC-CSM2-MR SSP5-8.5 (1)	10.22033/ESGF/CMIP6.3050 [11]
CanESM5 piControl (1)	10.22033/ESGF/CMIP6.3673 [12]
CanESM5 historical (20)	10.22033/ESGF/CMIP6.3610 [13]
CanESM5 SSP1-1.9 (5)	10.22033/ESGF/CMIP6.3682 [14]
CanESM5 SSP1-2.6 (20)	10.22033/ESGF/CMIP6.3683 [15]
CanESM5 SSP2-4.5 (20)	10.22033/ESGF/CMIP6.3685 [16]
CanESM5 SSP3-7.0 (19)	10.22033/ESGF/CMIP6.3690 [17]
CanESM5 SSP5-8.5 (8)	10.22033/ESGF/CMIP6.3696 [18]
CNRM-CM6-1-HR historical (1)	10.22033/ESGF/CMIP6.4067 [19]
CNRM-CM6-1-HR SSP1-2.6 (1)	10.22033/ESGF/CMIP6.4185 [20]
CNRM-CM6-1-HR SSP5-8.5 (1)	10.22033/ESGF/CMIP6.4225 [21]
EC-Earth3 piControl (1)	10.22033/ESGF/CMIP6.4842 [22]
EC-Earth3 historical (5)	10.22033/ESGF/CMIP6.4700 [23]
EC-Earth3 SSP1-1.9 (1)	10.22033/ESGF/CMIP6.4870 [24]
EC-Earth3 SSP1-2.6 (3)	10.22033/ESGF/CMIP6.4874 [25]
EC-Earth3 SSP2-4.5 (6)	10.22033/ESGF/CMIP6.4880 [26]
IPSL-CM5A2-INCA historical (1)	10.22033/ESGF/CMIP6.13661 [27]
IPSL-CM5A2-INCA SSP1-2.6 (1)	10.22033/ESGF/CMIP6.15711 [28]
IPSL-CM5A2-INCA SSP3-7.0 (1)	10.22033/ESGF/CMIP6.15714 [29]
MIROC6 historical (3)	10.22033/ESGF/CMIP6.5603 [30]
MIROC6 SSP1-1.9 (1)	10.22033/ESGF/CMIP6.5741 [31]
MIROC6 SSP1-2.6 (3)	10.22033/ESGF/CMIP6.5743 [32]
MIROC6 SSP2-4.5 (3)	10.22033/ESGF/CMIP6.5746 [33]
MIROC6 SSP3-7.0 (3)	10.22033/ESGF/CMIP6.5752 [34]
MIROC6 SSP5-8.5 (3)	10.22033/ESGF/CMIP6.5771 [35]
MIROC-ES2L historical (9)	10.22033/ESGF/CMIP6.5602 [36]
MIROC-ES2L SSP1-1.9 (9)	10.22033/ESGF/CMIP6.5740 [37]
MPI-ESM-1-2-HAM historical (3)	10.22033/ESGF/CMIP6.5016 [38]
MPI-ESM-1-2-HAM SSP3-7.0 (3)	no citable DOI
MPI-ESM1-2-LR piControl (1)	10.22033/ESGF/CMIP6.6675 [39]
MPI-ESM1-2-LR historical (45)	10.22033/ESGF/CMIP6.6595 [40]
MPI-ESM1-2-LR SSP1-1.9 (18)	10.22033/ESGF/CMIP6.6688 [41]
MPI-ESM1-2-LR SSP1-2.6 (46)	10.22033/ESGF/CMIP6.6690 [42]
MPI-ESM1-2-LR SSP2-4.5 (46)	10.22033/ESGF/CMIP6.6693 [43]
MPI-ESM1-2-LR SSP3-7.0 (48)	10.22033/ESGF/CMIP6.6695 [44]
MPI-ESM1-2-LR SSP5-8.5 (47)	10.22033/ESGF/CMIP6.6705 [45]
NorESM2-LM historical (3)	10.22033/ESGF/CMIP6.8036 [46]
NorESM2-LM SSP2-2.6 (1)	10.22033/ESGF/CMIP6.8248 [47]
NorESM2-LM SSP2-4.5 (3)	10.22033/ESGF/CMIP6.8253 [48]
NorESM2-LM SSP3-7.0 (3)	10.22033/ESGF/CMIP6.8268 [49]
NorESM2-LM SSP5-8.5 (1)	10.22033/ESGF/CMIP6.8319 [50]

Table S5 Data references for the CMIP6 models that met the selection criteria (see the Methods section 4) and are used for the analysis in this article. Numbers in parenthesis after the model name and type of run indicate the number of simulations of that type that were analyzed.

Supplementary References

[1] Auclair, G. & Tremblay, L. B. The role of ocean heat transport in rapid sea ice declines in the Community Earth System Model Large Ensemble. *J. Geophys. Res: Oceans* **123**, 8941–8957 (2018).

[2] Dix, M. *et al.* CSIRO-ARCCSS ACCESS-CM2 model output prepared for CMIP6 CMIP piControl (2019). URL https://doi.org/10.22033/ESGF/CMIP6.4311.

[3] Dix, M. *et al.* CSIRO-ARCCSS ACCESS-CM2 model output prepared for CMIP6 CMIP historical (2019). URL https://doi.org/10.22033/ESGF/CMIP6.4271.

[4] Dix, M. *et al.* CSIRO-ARCCSS ACCESS-CM2 model output prepared for CMIP6 ScenarioMIP SSP126 (2019). URL https://doi.org/10.22033/ESGF/CMIP6.4319.

[5] Ziehn, T. *et al.* CSIRO ACCESS-ESM1.5 model output prepared for CMIP6 ScenarioMIP SSP245 (2019). URL https://doi.org/10.22033/ESGF/CMIP6.4322.

[6] Dix, M. *et al.* CSIRO-ARCCSS ACCESS-CM2 model output prepared for CMIP6 ScenarioMIP ssp370 (2019). URL https://doi.org/10.22033/ESGF/CMIP6.4323.

[7] Dix, M. *et al.* CSIRO-ARCCSS ACCESS-CM2 model output prepared for CMIP6 ScenarioMIP SSP585 (2019). URL https://doi.org/10.22033/ESGF/CMIP6.4332.

[8] Wu, T. *et al.* BCC BCC-CSM2MR model output prepared for CMIP6 CMIP historical (2018). URL https://doi.org/10.22033/ESGF/CMIP6.2948.

[9] Xin, X. *et al.* BCC BCC-CSM2MR model output prepared for CMIP6 ScenarioMIP SSP126 (2019). URL https://doi.org/10.22033/ESGF/CMIP6.3028.

[10] Xin, X. *et al.* BCC BCC-CSM2MR model output prepared for CMIP6 ScenarioMIP SSP245 (2019). URL https://doi.org/10.22033/ESGF/CMIP6.3030.

[11] Xin, X. *et al.* BCC BCC-CSM2MR model output prepared for CMIP6 ScenarioMIP SSP585 (2019). URL https://doi.org/10.22033/ESGF/CMIP6.3050.

[12] Swart, N. C. *et al.* CCCma CanESM5 model output prepared for CMIP6 CMIP piControl (2019). URL https://doi.org/10.22033/ESGF/CMIP6.3673.

[13] Swart, N. C. *et al.* CCCma CanESM5 model output prepared for CMIP6 CMIP historical (2019). URL https://doi.org/10.22033/ESGF/CMIP6.3610.

[14] Swart, N. C. *et al.* CCCma CanESM5 model output prepared for CMIP6 ScenarioMIP SSP119 (2019). URL https://doi.org/10.22033/ESGF/CMIP6.3682.

[15] Swart, N. C. *et al.* CCCma CanESM5 model output prepared for CMIP6 ScenarioMIP SSP126 (2019). URL https://doi.org/10.22033/ESGF/CMIP6.3683.

[16] Swart, N. C. *et al.* CCCma CanESM5 model output prepared for CMIP6 ScenarioMIP SSP245 (2019). URL https://doi.org/10.22033/ESGF/CMIP6.3685.

[17] Swart, N. C. *et al.* CCCma CanESM5 model output prepared for CMIP6 ScenarioMIP ssp370 (2019). URL https://doi.org/10.22033/ESGF/CMIP6.3690.

[18] Swart, N. C. *et al.* CCCma CanESM5 model output prepared for CMIP6 ScenarioMIP SSP585 (2019). URL https://doi.org/10.22033/ESGF/CMIP6.3696.

[19] Voldoire, A. CNRM-CERFACS CNRM-CM6-1-HR model output prepared for CMIP6 CMIP historical (2019). URL https://doi.org/10.22033/ESGF/CMIP6.4067.

[20] Voldoire, A. CNRM-CERFACS CNRM-CM6-1-HR model output prepared for CMIP6 ScenarioMIP ssp126 (2020). URL https://doi.org/10.22033/ESGF/CMIP6.4185.

[21] Voldoire, A. CNRM-CERFACS CNRM-CM6-1-HR model output prepared for CMIP6 ScenarioMIP SSP585 (2019). URL https://doi.org/10.22033/ESGF/CMIP6.4225.

[22] EC-Earth Consortium (EC-Earth). EC-Earth-Consortium EC-Earth3 model output prepared for CMIP6 CMIP piControl (2019). URL https://doi.org/10.22033/ESGF/CMIP6.4842.

[23] (EC-Earth), E.-E. C. EC-Earth-Consortium EC-Earth3 model output prepared for CMIP6 CMIP historical (2019). URL https://doi.org/10.22033/ESGF/CMIP6.4700.

[24] (EC-Earth), E.-E. C. Ec-earth-consortium ec-earth3 model output prepared for cmip6 scenariomip ssp119 (2019). URL https://doi.org/10.22033/ESGF/CMIP6.4870.

[25] (EC-Earth), E.-E. C. EC-Earth-Consortium EC-Earth3 model output prepared for CMIP6 ScenariOMIP SSP126 (2019). URL https://doi.org/10.22033/ESGF/CMIP6.4874.

[26] (EC-Earth), E.-E. C. EC-Earth-Consortium EC-Earth3 model output prepared for CMIP6 ScenariOMIP SSP245 (2019). URL https://doi.org/10.22033/ESGF/CMIP6.4880.

[27] Boucher, O. *et al.* IPSL IPSL-CM5A2-INCA model output prepared for CMIP6 CMIP historical (2020). URL https://doi.org/10.22033/ESGF/CMIP6.13661.

[28] Boucher, O. *et al.* IPSL IPSL-CM5A2-INCA model output prepared for CMIP6 ScenarioMIP ssp126 (2020). URL https://doi.org/10.22033/ESGF/CMIP6.15711.

[29] Boucher, O. *et al.* IPSL IPSL-CM5A2-INCA model output prepared for CMIP6 ScenarioMIP ssp370 (2020). URL https://doi.org/10.22033/ESGF/CMIP6.15714.

[30] Tatebe, H. & Watanabe, M. MIROC MIROC6 model output prepared for CMIP6 CMIP historical (2018). URL https://doi.org/10.22033/ESGF/CMIP6.5603.

[31] Shiogama, H., Abe, M. & Tatebe, H. MIROC MIROC6 model output prepared for CMIP6 ScenarioMIP SSP119 (2019). URL https://doi.org/10.22033/ESGF/CMIP6.5741.

[32] Shiogama, H., Abe, M. & Tatebe, H. MIROC MIROC6 model output prepared for CMIP6 ScenarioMIP SSP126 (2019). URL https://doi.org/10.22033/ESGF/CMIP6.5743.

[33] Shiogama, H., Abe, M. & Tatebe, H. MIROC MIROC6 model output prepared for CMIP6 ScenarioMIP SSP245 (2019). URL https://doi.org/10.22033/ESGF/CMIP6.5746.

[34] Shiogama, H., Abe, M. & Tatebe, H. MIROC MIROC6 model output prepared for CMIP6 ScenarioMIP ssp370 (2019). URL https://doi.org/10.22033/ESGF/CMIP6.5752.

[35] Shiogama, H., Abe, M. & Tatebe, H. MIROC MIROC6 model output prepared for CMIP6 ScenarioMIP SSP585 (2019). URL https://doi.org/10.22033/ESGF/CMIP6.5771.

[36] Hajima, T. *et al.* MIROC MIROC-ES2L model output prepared for CMIP6 CMIP historical (2019). URL https://doi.org/10.22033/ESGF/CMIP6.5602.

[37] Tachiiri, K. *et al.* MIROC MIROC-ES2L model output prepared for CMIP6 ScenarioMIP SSP119 (2019). URL https://doi.org/10.22033/ESGF/CMIP6.5740.

[38] Neubauer, D. *et al.* HAMMOZ-Consortium MPI-ESM1.2-HAM model output prepared for CMIP6 CMIP historical (2019). URL https://doi.org/10.22033/ESGF/CMIP6.5016.

[39] Wieners, K.-H. *et al.* MPI-M MPI-ESM1.2-LR model output prepared for CMIP6 CMIP piControl (2019). URL https://doi.org/10.22033/ESGF/CMIP6.6675.

[40] Wieners, K.-H. *et al.* MPI-M MPI-ESM1.2-LR model output prepared for CMIP6 CMIP historical (2019). URL https://doi.org/10.22033/ESGF/CMIP6.6595.

[41] Milinski, S. *et al.* MPI-M MPI-ESM1.2-LR model output prepared for CMIP6 ScenarioMIP ssp119 (2019). URL https://doi.org/10.22033/ESGF/CMIP6.6688.

[42] Wieners, K.-H. *et al.* MPI-M MPI-ESM1.2-LR model output prepared for CMIP6 ScenarioMIP SSP126 (2019). URL https://doi.org/10.22033/ESGF/CMIP6.6690.

[43] Wieners, K.-H. *et al.* MPI-M MPI-ESM1.2-LR model output prepared for CMIP6 ScenarioMIP SSP245 (2019). URL https://doi.org/10.22033/ESGF/CMIP6.6693.

[44] Wieners, K.-H. *et al.* MPI-M MPI-ESM1.2-LR model output prepared for CMIP6 ScenarioMIP ssp370 (2019). URL https://doi.org/10.22033/ESGF/CMIP6.6695.

[45] Wieners, K.-H. *et al.* MPI-M MPI-ESM1.2-LR model output prepared for CMIP6 ScenarioMIP SSP585 (2019). URL https://doi.org/10.22033/ESGF/CMIP6.6705.

[46] Seland, y. *et al.* NCC NorESM2-LM model output prepared for CMIP6 CMIP historical (2019). URL https://doi.org/10.22033/ESGF/CMIP6.8036.

[47] Seland, y. *et al.* NCC NorESM2-LM model output prepared for CMIP6 ScenarioMIP ssp126 (2019). URL https://doi.org/10.22033/ESGF/CMIP6.8248.

[48] Seland, y. *et al.* NCC NorESM2-LM model output prepared for CMIP6 ScenarioMIP SSP245 (2019). URL https://doi.org/10.22033/ESGF/CMIP6.8253.

[49] Seland, y. *et al.* NCC NorESM2-LM model output prepared for CMIP6 ScenarioMIP ssp370 (2019). URL https://doi.org/10.22033/ESGF/CMIP6.8268.

[50] Seland, y. *et al.* NCC NorESM2-LM model output prepared for CMIP6 ScenarioMIP ssp585 (2019). URL https://doi.org/10.22033/ESGF/CMIP6.8319.
